# Multi-Classification Model for PPG Signal Arrhythmia Based on Time–Frequency Dual-Domain Attention Fusion

**DOI:** 10.3390/s25195985

**Published:** 2025-09-27

**Authors:** Yubo Sun, Keyu Meng, Shipan Lang, Pei Li, Wentao Wang, Jun Yang

**Affiliations:** 1 College of Electronic Information Engineering, Changchun University, Changchun 130022, China; yubo_sun6765@163.com (Y.S.); w2313736155@163.com (W.W.); 2 Jilin Provincial Key Laboratory of Human Health Status Identification and Function Enhancement, School of Electronic and Information Engineering, Changchun University, Changchun 130022, China; 3 Chongqing Institute of Green and Intelligent Technology, Chinese Academy of Sciences, Chongqing 400714, China; langshipan2837@163.com (S.L.); lipei1@cigit.ac.cn (P.L.)

**Keywords:** photoplethysmography (PPG), arrhythmia classification, deep learning, multi-domain attention network, wearable health monitoring

## Abstract

Cardiac arrhythmia is a leading cause of sudden cardiac death. Its early detection and continuous monitoring hold significant clinical value. Photoplethysmography (PPG) signals, owing to their non-invasive nature, low cost, and convenience, have become a vital information source for monitoring cardiac activity and vascular health. However, the inherent non-stationarity of PPG signals and significant inter-individual variations pose a major challenge in developing highly accurate and efficient arrhythmia classification methods. To address this challenge, we propose a Fusion Deep Multi-domain Attention Network (Fusion-DMA-Net). Within this framework, we innovatively introduce a cross-scale residual attention structure to comprehensively capture discriminative features in both the time and frequency domains. Additionally, to exploit complementary information embedded in PPG signals across these domains, we develop a fusion strategy integrating interactive attention, self-attention, and gating mechanisms. The proposed Fusion-DMA-Net model is evaluated for classifying four major types of cardiac arrhythmias. Experimental results demonstrate its outstanding classification performance, achieving an overall accuracy of 99.05%, precision of 99.06%, and an F1-score of 99.04%. These results demonstrate the feasibility of the Fusion-DMA-Net model in classifying four types of cardiac arrhythmias using single-channel PPG signals, thereby contributing to the early diagnosis and treatment of cardiovascular diseases and supporting the development of future wearable health technologies.

## 1. Introduction

Cardiovascular diseases (CVDs) remain a leading cause of mortality worldwide, with incidence rates continuing to rise in recent years. Among these, arrhythmia—characterized by abnormal heart rhythm and rate caused by disturbances in cardiac pacing and conduction—has become a major chronic condition posing significant public health risks. The 2024 World Health Organization (WHO) report states that non-communicable diseases (NCDs) have surpassed infectious diseases as the primary cause of global mortality, with cardiovascular diseases responsible for most NCD-related deaths [1]. Early detection and accurate classification of arrhythmias are essential for timely intervention and effective cardiovascular disease management. Conventional arrhythmia monitoring methods rely mainly on clinical tools such as 12-lead electrocardiograms (ECGs) and 24 h Holter monitors [2,3,4,5]. Although highly accurate, these methods require specialized equipment and manual interpretation, making them unsuitable for long-term, real-time monitoring in large populations. Furthermore, ECG electrodes require direct skin contact, and multiple leads can cause discomfort and interfere with daily activities, limiting their feasibility for continuous wearable monitoring. In recent years, advances in artificial intelligence and wearable technology have brought increasing attention to photoplethysmography (PPG)-based arrhythmia monitoring. Due to its non-invasive nature, low cost, and simplicity, PPG has emerged as a promising alternative to ECG for heart rhythm monitoring. Calvert et al. [6] found that over 90% of participants in a remote atrial fibrillation (AF) monitoring study using smartphone applications considered the PPG-based system easy to operate and suitable for daily self-monitoring. Classification algorithms play a central role in PPG-based arrhythmia detection.

With continued advances in artificial intelligence (AI), researchers have developed diverse photoplethysmography (PPG)-based arrhythmia classification models to improve detection accuracy and support real-time monitoring. These models generally fall into two categories: traditional machine learning (ML) and deep learning (DL) approaches. Conventional ML approaches typically involve three stages: data preprocessing, handcrafted feature extraction, and classifier training. Because PPG signals are susceptible to noise and motion artifacts, acquiring high-quality signals is essential. Feature extraction directly affects classifier performance and remains the most critical step in ML-based classification. Commonly used classifiers include support vector machines (SVMs) [7,8,9,10], random forests (RFs) [11,12], decision trees (DTs) [13], and artificial neural networks (ANNs) [14,15]. Qananwah et al. [16] enhanced classification performance by integrating principal component analysis (PCA) with PPG-based ML algorithms. However, ML methods rely heavily on domain expertise for feature engineering, risking the omission of critical information and exhibiting limited generalization when processing high-dimensional, complex sequential data. In contrast, AI-augmented classification strategies are essential for improving accuracy, scalability, and deployment on wearable or edge devices. Deep learning models remove the need for manual feature extraction through end-to-end learning and automatically identify meaningful patterns in PPG signals, substantially enhancing classification robustness.

Recently developed models, including bidirectional long short-term memory (Bi-LSTM) networks, residual networks (ResNet), and Transformer architectures, have shown superior performance in time-series modeling, noise robustness, and computational efficiency. Most deep learning (DL)-based arrhythmia studies focus on single-type detection tasks, such as atrial fibrillation (AF) identification. For example, Shashikumar et al. [17] developed a convolutional neural network (CNN)-based model that transforms PPG signals into two-dimensional spectrograms and incorporates signal quality indices, achieving an accuracy of 91.8%. Aliamiri and Shen [18] proposed a convolutional recurrent neural network (CRNN) architecture that combines CNN and recurrent neural network (RNN) components with multimodal signal quality control, attaining an accuracy of 98.19% across 19 AF subjects. Cheng et al. [19] used time–frequency representations as inputs to a CNN–long short-term memory (LSTM) hybrid model, achieving performance metrics exceeding 98%. Beyond classification, researchers have investigated denoising and label quality enhancement for PPG signals. Mohagheghian et al. [20] employed a convolutional denoising autoencoder (CDA) to reconstruct noisy PPG inputs, achieving an AF detection accuracy of 91.02%. To address low-quality PPG annotations, Ding [21] proposed a cluster member consistency (CMC) loss function to mitigate label noise and enhance model generalizability. In terms of form factor, Van Steijn et al. [22] investigated a ring-based PPG sensor for ventricular arrhythmia detection, achieving 94% sensitivity and demonstrating its viability for specific arrhythmia types. Compared with single-type arrhythmia detection, multi-type arrhythmia classification remains relatively underexplored. These models aim to identify and differentiate multiple rhythm types simultaneously. Liu et al. [23] used a VGGNet-16 architecture to classify six rhythm types from 10 s PPG segments, achieving 85.0% overall accuracy and an area under the curve (AUC) of 0.978. Wu et al. [24] proposed a hybrid ResNet–BiLSTM model that jointly encodes spatial and temporal features from PPG, achieving accuracy above 92%. Liu et al. [25] introduced a CNN–Transformer architecture to capture both local and global dependencies, achieving an F1 score of 86.8%. Similarly, Gelen et al. [26] combined wavelet transforms with quantum-inspired feature extraction, increasing multi-class classification accuracy to 91.30%. Real-world studies also confirm the potential of PPG-based wearables. Chen et al. [27] employed a DenseNet architecture, achieving sensitivity and specificity above 94%. Bulut et al. [28] used a one-dimensional (1D) CNN to classify normal sinus rhythm (NSR), AF, and premature atrial contraction (PAC) rhythms from wearable PPG data, achieving 95.17% accuracy and reinforcing the effectiveness of deep CNNs for PPG analysis. Deep learning–based PPG arrhythmia classification offers clear advantages in automation, real-time capability, and wearable deployment, enabling practical solutions for personalized and home-based cardiac health monitoring.

In this study, we propose a novel deep learning framework for arrhythmia classification using single-channel photoplethysmography (PPG) signals. The framework employs a dual-branch architecture to extract complementary time-domain and frequency-domain features across multiple scales. It incorporates self-attention mechanisms and cross-domain fusion modules to enhance feature representation and improve model robustness. The proposed framework is evaluated on a publicly available dataset and comprehensively compared with state-of-the-art deep learning architectures. Multiple evaluation metrics are employed to assess classification performance, stability, and real-time applicability, offering new insights and practical references for PPG-based intelligent arrhythmia monitoring systems.

## 2. Materials and Methods

### 2.1. Overview of the Proposed Model

**Figure 1:**
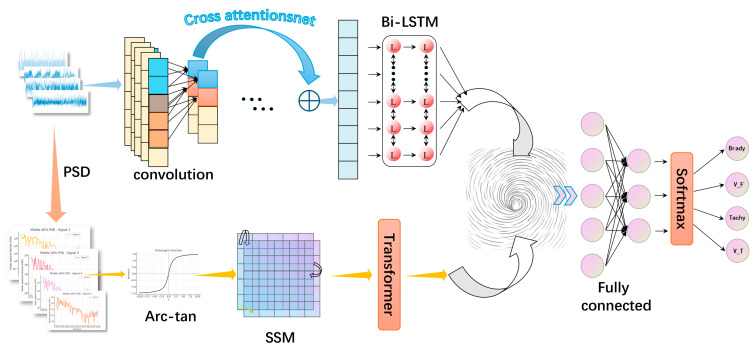
Overall architecture of Fusion-DMA-Net. Blue and orange arrows indicate data flow directions in the time domain and frequency domain branches, respectively. The red circular nodes in the Bi-LSTM emphasize bidirectional data flow. The spiral pattern represents the feature fusion module.

This study proposes a Fusion Dual-domain Multi-Attention Network (Fusion-DMA-Net) designed for multi-class arrhythmia detection based on photoplethysmography (PPG) signals. The model adopts a parallel dual-branch architecture that jointly processes time-domain and frequency-domain features, aiming to extract discriminative patterns across multiple temporal and spectral scales and thereby enhance classification performance across various arrhythmic rhythms. As illustrated in Figure 1, the overall framework consists of four core modules: a data preprocessing module, a multiscale feature extraction module, a feature fusion module, and a classification module.

In the time-domain branch, a convolutional neural network (CNN) is combined with a bidirectional long short-term memory (BiLSTM) network to construct the feature backbone. A novel cross-scale residual attention (CSRA) mechanism is further integrated to enhance the model’s ability to capture rhythm discontinuities and waveform dynamics. In parallel, the frequency-domain branch employs nonlinear transformation (arc-tangent) and self-similarity matrix (SSM) construction, followed by a Transformer encoder to model long-range dependencies and global contextual features within the spectral domain. This dual-branch design enables the network to learn complementary information from both time and frequency perspectives. During feature fusion, the model incorporates a hierarchical channel attention mechanism and cross-domain interactive attention to enable deep interaction between branches. A gating mechanism is used to adaptively fuse features from different sources, thereby improving the overall discriminative capability. Finally, the integrated representation is passed through fully connected layers for automatic multi-class arrhythmia classification.

### 2.2. Dual-Domain Feature Extraction

To fully capture the multiscale and multi-domain characteristics of PPG signals, a dual-branch feature extraction module is designed, composed of time-domain and frequency-domain sub-networks. These two branches focus, respectively, on modeling rhythmic dynamics in the temporal dimension and energy distribution patterns in the frequency domain, thereby enhancing the model’s accuracy and robustness in arrhythmia recognition.

#### 2.2.1. Time-Domain Feature Extraction

**Figure 2:**
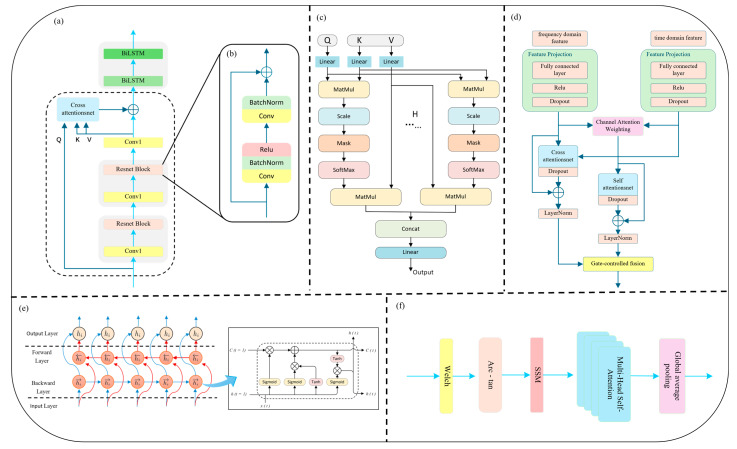
Structure of the residual block in the time-domain branch: (**a**) Full pipeline of time-domain feature extraction; (**b**) Structure of the residual block in the time-domain branch; (**c**) Schematic of the interactive attention mechanism; (**d**) Architecture of the multi-domain feature fusion module; (**e**) Architecture of the CSIA module with multi-head attention and residual inputs; (**f**) Full pipeline of frequency-domain feature extraction.

In the time-domain branch, we introduce a novel Cross-Scale Residual Attention (CSRA) mechanism to enhance the network’s sensitivity to rhythm variations and mitigate the limitations of conventional deep convolutional structures in modeling long-range dependencies and multi-scale temporal patterns. In the field of deep learning, the traditional CNN + LSTM architecture has been widely used for temporal sequence modeling, with CNNs excelling at capturing local structures and LSTMs handling sequential dependencies. However, CNNs typically have limited receptive fields, and deep stacking can lead to vanishing gradients. Meanwhile, standard LSTM architectures are insufficient in capturing bidirectional rhythm dynamics, thus limiting their ability to model complex arrhythmic behavior. To address these challenges, we incorporate a residual network backbone with skip connections that enable direct information flow between shallow and deep layers. This alleviates gradient degradation, accelerates convergence, and enables deeper network depth without performance loss. Each residual block consists of two 1D convolutional layers, combined with Batch Normalization [29] and ReLU activation [30], forming a multi-scale learning unit that preserves both low-level detail and high-level abstraction (see Figure 2b).

To further enhance temporal contextual modeling, an Interactive Attention Mechanism is integrated atop the residual structure. This mechanism is designed to model the relationships between two distinct sequences by allowing one (the query) to attend to another (the key-value pair), thus enabling dynamic cross-time information fusion and feature reweighting. The detailed computation is defined in Equation (1):

(1) $$\mathrm{Attention}\left(Q,K,V\right)=\mathrm{Softmax}\left(\frac{QK^{T}}{\sqrt{d_{k}}}\right)\times V$$

Here, Q, K, and V represent the query, key, and value matrices, respectively. The term $d_{k}$ denotes the dimensionality of the key vectors. The product $QK^{T}$ computes the dot-product similarity between the queries and keys, resulting in an attention score matrix. Unlike standard self-attention, in which the queries, keys, and values are derived from the same sequence, the formulation in this module allows the queries (Q) to originate from one sequence, while the keys (K) and values (V) are drawn from another, enabling cross-sequence interaction and information alignment.

Building upon the above, this study further introduces Multi-Head Attention (MHA) (as illustrated in Figure 2c), combined with Layer Normalization, to enhance multi-scale temporal modeling. The underlying principles are detailed in Equations (2)–(4). The multi-head attention mechanism enables parallel alignment and aggregation of multimodal features across different subspaces. Each attention head autonomously attends to distinct temporal fragments or frequency bands, improving the model’s sensitivity to various arrhythmic rhythm disruptions. The residual connection ensures smooth information flow, while LayerNorm stabilizes gradient propagation and prevents excessive fitting or degeneration, thus achieving incremental feature enhancement. In this work, we designate the deep residual features as both query (Q) and key (K), while the adaptively reconstructed input serves as the value (V), jointly forming a Cross-Scale Interactive Attention (CSIA) module. This structure effectively reinforces the fusion between low-level information and high-level semantic representations.

(2) $${\mathrm{head}}_{i}=\mathrm{Attention}\left(QW_{i}^{Q},KW_{i}^{K},VW_{i}^{V}\right)\;i\in \left[1,H\right]$$

Here, ${head}_{i}$ denotes the output vector of the i attention head, which captures localized feature representations extracted by that head. The function Attention refers to the scaled dot-product attention mechanism. The matrices $W_{i}^{Q}$, $W_{i}^{K}$, and $W_{i}^{V}$ are the learnable linear projection weights corresponding to the i head, applied to the query (Q), key (K), and value (V), respectively, to map them into distinct subspaces. The parameter H denotes the total number of attention heads.

(3) $$\mathrm{MultiHeadAttention}\left(Q,K,V\right)=\mathrm{Concat}\left({\mathrm{head}}_{1},{\mathrm{head}}_{2},\ldots ,{\mathrm{head}}_{H}\right)\;W^{O}$$

Here, $Concat\left(\cdot \right)$ denotes the concatenation operation, which merges the output feature vectors from all attention heads (${head}_{1}$ to ${head}_{H}$) into a single, larger vector. The matrix $W^{O}$ is a linear projection matrix applied to the concatenated features, reducing the dimensionality to the desired output space.

(4) $$Y=\mathrm{LayerNorm}\left(\mathrm{MultiHeadAttention}\left(Q,K,V\right)+X\right)$$

Here, Y denotes the final output matrix or tensor. $LayerNorm\left(\cdot \right)$ refers to the layer normalization operation, which stabilizes training by normalizing intermediate activations. X represents the input tensor to the residual connection.

To address the periodic and non-stationary nature of arrhythmia signals, a Bidirectional LSTM (BiLSTM) layer is employed following the attention module (Figure 2e). Compared to standard LSTM, BiLSTM captures both past and future dependencies, enhancing modeling of complex rhythms such as atrial fibrillation (AF) and premature ventricular contractions (PVC). Its bidirectional feedback loop improves model robustness against signal interruptions and local noise. As shown in Figure 2a, the PPG signal is first passed through a 1D causal convolution and max-pooling layer for preliminary feature extraction and downsampling. Two stacked residual blocks then encode deep representations. The original input is reconstructed via global average pooling and a projection layer to serve as the attention Value input. Finally, the output is passed through two BiLSTM layers to capture long-term dependencies and construct a robust time-domain representation for subsequent classification.

#### 2.2.2. Frequency-Domain Feature Extraction

In the analysis of arrhythmias using PPG signals, time-domain features primarily focus on waveform morphology and rhythm variability, such as local fluctuations in R–R intervals, which can effectively reflect irregularities in cardiac rhythms. However, certain types of arrhythmias—such as atrial flutter, high-frequency fibrillations, or harmonic ventricular tachycardia—exhibit persistent and latent spectral patterns that are difficult to capture through time-domain analysis alone. To address this limitation, a dedicated frequency-domain analysis branch is introduced in this study, aiming to extract energy distribution characteristics and spectral stability features. This perspective supplements the time-domain representation, especially in scenarios involving high-frequency disturbances or chronic rhythm disorders. Frequency-domain modeling enables the identification of latent rhythmic structures within PPG signals and facilitates multi-scale rhythm detection. For instance, although atrial fibrillation appears disorganized in the time domain, it often presents as sustained high-frequency energy in the 4–7 Hz band of the frequency spectrum. Different arrhythmic types exhibit distinct characteristics across low-frequency (LF, 0.04–0.15 Hz), high-frequency (HF, 0.15–0.4 Hz), and even higher bands. Frequency-domain networks allow for the parallel monitoring of multiple frequency bands, offering more fine-grained contrast in rhythm intensity than time-domain models.

To effectively model such frequency-specific features, this study proposes a frequency-domain feature extraction branch that integrates Transformer and BiLSTM architectures with arc-tangent nonlinear mapping and self-similarity matrix (SSM) encoding. The overall architecture of the frequency-domain branch is illustrated in Figure 2f. The proposed method adopts a spectral self-similarity input strategy, in which a self-similarity matrix (SSM) defined as $S=X{\cdot X}^{T}$ is introduced as the primary input to the network. Based on standard sinusoidal positional encoding, an additional lightweight convolutional module is appended to perform dynamic adjustment of positional embeddings, allowing the model to learn the superimposed relationships between low-frequency modulations (e.g., respiratory sinus arrhythmia) and high-frequency components within the PPG signal. Specifically, Welch’s Power Spectral Density (PSD) estimation method is employed to decompose the PPG signal into its spectral components. Unlike direct application of the Fast Fourier Transform (FFT), Welch’s method enhances the stability of spectral estimates by reducing the variance through windowing, segment overlap, and averaging operations.

Welch’s method is a classical approach for PSD estimation widely used in signal processing to obtain a smoother and lower-variance power spectrum. The original signal $x\left[n\right]$ of length N is divided into K overlapping or non-overlapping segments, each of length L. Each segment $x_{k}\left[n\right]$ is then multiplied by a window function $w\left[n\right]$. The periodogram of each segment is calculated as follows (Equation (5)):

(5) $$P_{k}\left(f\right)=\frac{1}{U}{\left|\mathrm{FFT}\{x_{k}\left[n\right]\cdot w\left[n\right]\}\right|}^{2}\;\;n\in [0,\;L-1]$$

Here, $U=\frac{1}{L}\;\sum_{n=0}^{L-1}\;w^{2}\left[n\right]$ is the normalization factor representing the window energy. $P_{k}\left(f\right)$ denotes the power spectral estimate of the k segment. $x_{k}\left[n\right]$ is the n sample from the k signal segment. FFT{⋅} refers to the Discrete Fourier Transform (DFT) operation.

Finally, the periodograms of all K segments are averaged pointwise along the frequency axis to obtain the final power spectral estimate, as defined in Equation (6):

(6) $$P_{\mathrm{Welch}}\left(f\right)=\frac{1}{K}\;\sum_{k=1}^{K}{\;P}_{k}\left(f\right)$$

Here, K denotes the total number of segments, determined by the original signal length N, segment length L, and overlap ratio. The averaging operation significantly reduces the variance of individual periodograms, resulting in a smoother and more stable spectral estimate. The specific parameters and methodologies of the proposed model are described in detail in the Supplementary Materials.

Subsequently, an arc-tangent nonlinear mapping is applied to compress the PSD values from the original domain [0, +∞) into the bounded interval (−π/2, +π/2). This transformation mitigates the impact of local energy spikes (e.g., caused by motion artifacts), while preserving the relative energy ranking across frequency bands, thereby enhancing the model’s robustness and sensitivity in frequency-domain representation. The compressed spectrum is further transformed into a Self-Similarity Matrix (SSM) by computing the inner product between spectral vectors. Each element (i, j) in the resulting matrix represents the energy similarity between frequency bands i and j over the entire signal segment. This operation effectively lifts the original 1D spectral structure into a 2D similarity map, emphasizing structural relationships among frequency bands. It enables the model to intuitively capture rhythmic patterns such as spectral resonance, inter-frequency transitions, and repetitive spectral motifs, thereby enhancing the representation of spectral heterogeneity and multi-scale periodicity.

On top of the SSM, a Transformer encoder is introduced to perform deep feature modeling using multi-head self-attention. In this study, four attention heads are employed in parallel to learn non-local dependencies between spectral blocks in different subspaces. This design effectively breaks through the receptive field limitations of local-window methods commonly used in spectral modeling. The output of the Transformer is combined with the original spectral input via residual connections and layer normalization, facilitating stable long-range dependency modeling. A 1 × 1 convolutional layer is subsequently applied to project the channel dimension of the attention output. This layer maintains the lightweight nature of the network while performing cross-channel linear feature fusion. Finally, a bidirectional Long Short-Term Memory (BiLSTM) network is adopted to further model the sequential dynamics along the frequency axis. This architecture is capable of capturing rhythmic transitions such as enhancement–suppression–reemergence across frequency bands, making it especially suitable for detecting complex arrhythmias like atrial fibrillation, high-frequency flutter, and ventricular fibrillation, which often involve frequency drift and localized modulation.

The proposed frequency-domain feature extraction strategy centers on structural similarity modeling and sequential dependency learning. By combining dynamic range compression, structured SSM representation, multi-head attention, and BiLSTM sequence learning, the method systematically extracts multi-scale rhythmic information in the spectral domain of PPG signals. Compared to fully stacked Transformer architectures, this design achieves a favorable trade-off between model capacity and computational efficiency. Coupled with residual connections, dropout regularization, and layer normalization, the model demonstrates strong generalization and anti-overfitting capabilities, making it well-suited for real-time rhythm anomaly detection in wearable PPG applications.

### 2.3. Feature Fusion

The feature fusion module serves as a critical component within the Fusion-DMA-Net architecture, designed to unify and enhance deep representations from the temporal and frequency branches. Its goal is to improve the model’s discriminative power in classifying various types of arrhythmias. As illustrated in Figure 2d, this module consists of four major components: feature alignment, channel attention weighting, cross-domain interaction, and a gated fusion mechanism.

To address the inconsistency in sequence length and channel dimension between the temporal and frequency-domain outputs, the feature projection layer is first introduced to map both branches into a unified feature space. For sequence alignment, 1D upsampling and zero-padding are applied to ensure temporal compatibility across both feature domains, thereby facilitating subsequent fusion operations. Next, to emphasize the discriminative components in the fused features, a channel attention mechanism is applied independently to both temporal and frequency-domain features. This mechanism evaluates the relative importance of each channel by analyzing its global activation, enabling the model to enhance task-relevant features while suppressing irrelevant or redundant ones. The result is a more robust and informative joint representation. In the deep fusion stage, a Hybrid Cross–Self Attention Mechanism is proposed. Specifically:

- The cross-attention module treats the frequency-domain features as the query (Q), and the temporal-domain features as the key (K) and value (V), enabling global dependency modeling across modalities. This allows the model to learn how spectral characteristics modulate temporal patterns.
- The self-attention module, in contrast, operates within the fused feature space to capture long-range dependencies and reinforce contextual coherence between internal elements.

To dynamically balance the contributions from these two attention pathways, a gating mechanism is introduced. Inspired by gated recurrent units (GRU), this mechanism adaptively merges the outputs of the cross- and self-attention modules via a learnable gating unit. It effectively controls the flow of information, ensuring that the fused representation is both comprehensive and non-redundant. The final high-level fused features are passed through a classification head, consisting of a flatten layer, two fully connected layers, and a Softmax layer that outputs the predicted probability distribution across arrhythmia classes. By jointly modeling multi-type rhythm patterns and deeply integrating high-dimensional features, the classification capability and precision of the network are significantly enhanced.

This multi-domain fusion strategy enables the effective integration of time- and frequency-domain representations, enhancing global modeling and discriminative ability while maintaining a lightweight architecture. It not only enables cooperative modeling of heterogeneous information sources but also offers a robust solution for accurate arrhythmia detection in complex PPG signal scenarios—highlighting its strong clinical potential and algorithmic scalability.

## 3. Experiments

### 3.1. Data Source

In this study, the photoplethysmography (PPG) waveform data used for training, validation, and testing were obtained from the publicly available PhysioNet/Computing in Cardiology Challenge 2015 database [31]. In public datasets, the 2015 PhysioNet Challenge remains the only source that provides expert-validated true positive PPG signals of potentially life-threatening arrhythmic events. This database focuses on five categories of arrhythmias with potential life-threatening implications, as defined in Table 1, including asystole, extreme bradycardia, extreme tachycardia, ventricular tachycardia (VT), and ventricular flutter/fibrillation (VF).

The dataset was collected from bedside monitoring systems in intensive care units (ICUs), comprising a total of 750 original recordings. Each record contains two ECG leads and at least one continuous 5 min segment of either PPG or arterial blood pressure (ABP) waveform data. Each waveform is annotated with alarm labels that indicate whether a true arrhythmic event occurred. For the purposes of this study, only samples containing true positive alarm annotations and available PPG waveforms were included in the analysis. This study included only samples with true positive alarm records and PPG waveforms. Considering the absence of discernible features in asystole cases, only the four PPG-based arrhythmia subtypes were retained, resulting in a total of 92 valid cases. Details of data acquisition and label definitions can be found in the original publication by [31]. (https://physionet.org/content/challenge-2015/1.0.0/ (accessed on 24 September 2025)).

### 3.2. Preprocessing

The PhysioNet Challenge 2015 dataset was released after initial integration and basic preprocessing. To further eliminate residual low-frequency drift and high-frequency noise, and to enhance the stability of frequency-domain features, a fourth-order Butterworth bandpass filter (0.05–30 Hz) was first applied to remove high-frequency noise and low-frequency baseline wander. This was followed by a moving average filter to smooth the signal. Following filtering, a moving average filter was applied to smooth the signal. To further suppress baseline wander, a hybrid multi-scale correction method combining wavelet transform and cubic spline interpolation was adopted. This approach effectively removes both global drift and local trend fluctuations, thereby enhancing signal stability and preserving the underlying periodic structure. Finally, all signals were scaled to the [0, 1] range through min–max normalization, ensuring consistent feature scaling for subsequent model input. However, we also acknowledge that additional filtering may introduce phase delays or signal edge distortions, particularly at points of abrupt rhythm changes or peak boundaries. To minimize phase distortion, we employed zero-phase filtering using the filtfilt method in our preprocessing pipeline.

### 3.3. Sample Partitioning and Data Augmentation

To address sample partitioning and data augmentation while avoiding potential data leakage, we first performed stratified splitting of the dataset by arrhythmia type into training, validation, and test sets with a ratio of 6:2:2. This ensured balanced representation of all classes across the subsets, preventing the omission of minority classes in the test set and minimizing the risk of data leakage during subsequent augmentation. Given the inherent class imbalance among different arrhythmia types, a stratified sampling and weight-aware sliding window augmentation strategy was designed. A time-series augmentation approach based on a sliding window was then applied, using a window length of 10 s. The window stride was dynamically adjusted according to the class-specific weight distribution in the dataset, enabling a class-weight-guided data expansion strategy. This method preserves the structural integrity of the signals while effectively mitigating the impact of class imbalance on model training, thereby enhancing the model’s generalization capability. Specifically, the number of original segments for each class was first counted to calculate their relative weights. Based on the class-specific weight $\tilde{w_{c}}$ the sliding window stride was then determined as follows:

(7) $$S_{c}=\mathrm{clip}\left(\left\lfloor \frac{S_{\mathrm{ref}}}{\tilde{w_{c}^{\beta }}}\right\rfloor ,S_{\min},S_{\max}\right)$$

Here, $S_{\mathrm{ref}}=250$ denotes the reference stride (1 s), while $S_{min}=125$, $S_{max}=500$ define the lower and upper bounds of the stride (corresponding to 0.5–2 s). The parameter β=1.0 serves as a tuning factor. Rarer classes are assigned smaller strides to generate more augmented samples, whereas more abundant classes use larger strides to reduce redundancy. In this study, the target arrhythmias include bradycardia, tachycardia, ventricular flutter/fibrillation (VFB), and ventricular tachycardia (VTA).

### 3.4. Evaluation Metrics

To systematically evaluate the classification performance of the proposed model, all metrics in this study were calculated based on the confusion matrix, using the number of true positives (TP), false positives (FP), true negatives (TN), and false negatives (FN). The following evaluation metrics were employed for each arrhythmia class: Precision (Pre), Sensitivity (Sen), Specificity (Spe), F1-score, and Accuracy (Acc). The definitions and formulas for each metric are listed below:

1. Precision (Pre): The proportion of correctly predicted positive samples among all predicted positive samples, as shown in Equation (7):
  (8) $$Pre=\frac{TP}{TP+FP}\times 100\%$$
2. Sensitivity (Sen): The proportion of actual positive samples that are correctly predicted as positive, as shown in Equation (8):
  (9) $$Sen=\;\frac{TP}{TP+\;FN}\;\times 100\%$$
3. Specificity (Spe): The proportion of actual negative samples that are correctly predicted as negative, as shown in Equation (9):
  (10) $$Spe=\;\frac{TN}{FP+\;TN}\;\times 100\%$$
4. F1 score: The harmonic mean of precision and recall (sensitivity), as shown in Equation (10):
  (11) $$F1\;score\;=\;\frac{Precision\;\times Recall}{Precision+\;Recall}\;\times 100\%$$
5. Accuracy (Acc): The proportion of correctly classified samples among the total number of samples, as shown in Equation (11):
  (12) $$Acc=\;\frac{TP+TN}{TP+\;FN+FP+TN}\;\times 100\%$$

### 3.5. Experimental Setup

All experiments were implemented in Python 3.11, based on the TensorFlow 2.14 deep learning framework. Model training and evaluation were conducted on a server equipped with the following hardware specifications: 8-core vCPU, 30 GiB RAM, and a single NVIDIA A10 GPU. The operating system was Ubuntu 22.04, with CUDA version cu118.

During training, the model was optimized using the Adam optimizer [32] with an initial learning rate of 0.0005, and the Kullback–Leibler (KL) divergence was employed as the loss function. The batch size was set to 60, and the total number of training epochs was 160. To improve training stability and prevent overfitting, early stopping and a ReduceLROnPlateau callback were adopted. Specifically, the learning rate was reduced by a factor of 10 if the validation loss did not improve for 30 consecutive epochs. To address class imbalance, class weighting was applied during training. All network layers were initialized using the He normal initializer [33], and Dropout with a rate of 0.2 was incorporated into key layers to enhance model generalization. The best-performing model on the validation accuracy was saved using model checkpointing for final evaluation and inference.

## 4. Results and Discussion

### 4.1. Training and Validation Performance

**Figure 3:**
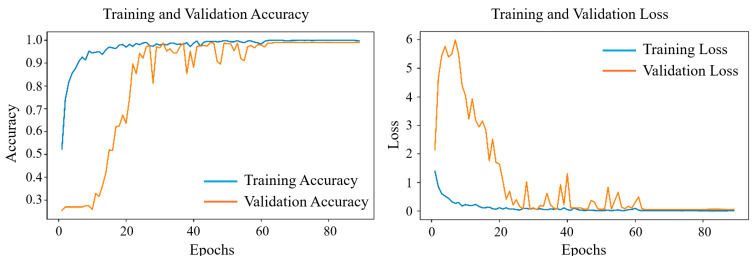
Performance evaluation of the Fusion-DMA-Net and ablation models: (**a**) Training and validation accuracy curves; (**b**) Training and validation loss curves.

During training, the Fusion-DMA-Net model was trained with a batch size of 60 and an initial learning rate of 0.0005. The Kullback–Leibler divergence (KLDivergence) was employed as the loss function to penalize deviations between the predicted and true probability distributions. The model was optimized using the Adam optimizer, which adaptively adjusts the learning rate based on the first and second moments of past gradients, thereby accelerating convergence and improving adaptability to various loss surfaces. A learning rate decay strategy was incorporated, and a ModelCheckpoint callback was used to monitor validation accuracy (val_accuracy) and retain the best-performing model. The training and validation learning curves of Fusion-DMA-Net are illustrated in Figure 3, demonstrating favorable convergence behavior and strong robustness.

To prevent overfitting during training, multiple regularization and generalization enhancement techniques were incorporated into both the model architecture and training process. Specifically, Dropout with a rate of 0.2 was applied to the BiLSTM and fully connected layers, while SpatialDropout was introduced in the residual layers to prevent excessive co-adaptation of neurons. In addition, an Early Stopping strategy was employed to halt training if the validation loss failed to improve over consecutive epochs, thereby reducing the risk of overfitting in later stages of training. During training, the ReduceLROnPlateau strategy was employed to dynamically adjust the learning rate, allowing for automatic reduction when model performance plateaued, thereby promoting a smoother convergence process. On the data level, a class-weight-aware sliding window (WASW) augmentation method was applied to densely sample and expand minority class instances in the training set. Additionally, class weights were incorporated into the loss function to strengthen the learning of underrepresented classes, effectively mitigating the impact of class imbalance. In terms of model architecture, a multi-branch design with time–frequency decoupled modeling and attention-based fusion mechanisms was introduced to achieve modular information flow construction. This not only enhances the model’s representational capacity but also reduces feature redundancy, thereby improving stability and generalization. Furthermore, all training, validation, and test data were strictly partitioned to ensure complete data isolation and prevent sample leakage, avoiding false generalization effects. Despite the integration of multiple structural modules, the total number of parameters remains controlled at approximately 1.46 million, resulting in a more lightweight architecture compared to conventional large-scale CNN models and reducing the risk of overfitting due to parameter redundancy. The synergistic effect of the above strategies significantly enhances the model’s robustness and generalization across different arrhythmia categories, ensuring both the stability and effectiveness of the training process.

As shown in the training accuracy curve (Figure 3a), the model starts with an initial training accuracy of approximately 35% at Epoch 1, rapidly increasing to over 90% within the first 10 epochs. After Epoch 20, the accuracy gradually approaches 99%, exhibiting a stable and smooth convergence trend throughout the training process. The validation accuracy also rises from an initial level of approximately 30%, showing a clear upward trend between Epochs 10 and 30. Although some fluctuations are observed during this period, the overall trajectory remains consistently positive. By around Epochs 70 to 80, the validation accuracy begins to plateau, approaching the training accuracy level, which indicates good convergence and strong generalization capability. The corresponding loss curve (Figure 3b) exhibits a typical optimization pattern characterized by an initial high-loss phase, followed by rapid decline, mid-phase fluctuations, and eventual stabilization. The training loss starts at approximately 1.0 and drops sharply to below 0.1 within the first 15 epochs, after which it gradually decreases and stabilizes around 0.02. In contrast, the validation loss shows considerable fluctuation during the first 1–10 epochs, reaching a peak of approximately 6.0, but progressively converges toward 0.1 in the later stages, indicating improved model stability and generalization. Between Epochs 20 and 60, several sharp spikes are observed in the validation loss curve. However, we argue that this phenomenon does not indicate typical overfitting behavior. First, during this period, the validation accuracy remains consistently high without any noticeable decline, suggesting that the model’s generalization capability remains stable. Second, these transient increases in loss are more likely attributed to normal optimization dynamics, such as fluctuations introduced by mini-batch training, variance induced by Dropout regularization, or temporary instability following learning rate adjustments triggered by the scheduler. More importantly, after Epoch 60, both the training and validation loss curves converge to a stable and highly consistent pattern, with accuracy approaching saturation. This indicates that the model has achieved robust convergence, and there is no need to further extend the number of training epochs.

### 4.2. Performance on the Test Set

#### 4.2.1. Confusion Matrix

**Figure 4:**
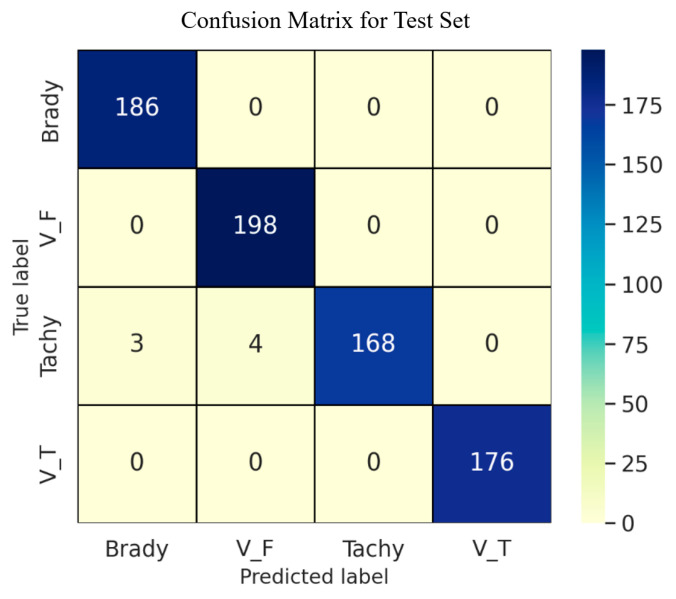
Confusion matrix of Fusion-DMA-Net on the test set.

As shown in Figure 4, the confusion matrix summarizes the classification results of Fusion-DMA-Net on the test dataset. Diagonal elements indicate correctly classified samples for each arrhythmia category. The model achieves an overall classification accuracy of 99.05%, demonstrating strong generalization performance. Specifically, only a small number of samples were misclassified across all four rhythm types. Both true positive (TP) and true negative (TN) counts approach the total number of samples, with an overall misclassification rate below 1%. In terms of inter-class confusion, three samples of Tachycardia were misclassified as Ventricular Flutter/Fibrillation (VF), accounting for approximately 2.4% of the Tachycardia subset. All other misclassifications were extremely limited. Notably, the Bradycardia, Ventricular Flutter/Fibrillation, and Ventricular Tachycardia (VT) classes exhibited zero false positives or false negatives, reflecting the model’s strong robustness and consistent classification ability for these rhythm types. The confusion matrix confirms that the model maintains a high level of classification accuracy across all four arrhythmia categories, despite varying inter-class similarities. Notably, it demonstrates stable and reliable performance in identifying clinically critical rhythms such as ventricular tachycardia (V_T) and ventricular fibrillation (V_F).

#### 4.2.2. ROC & PR Curves

**Figure 5:**
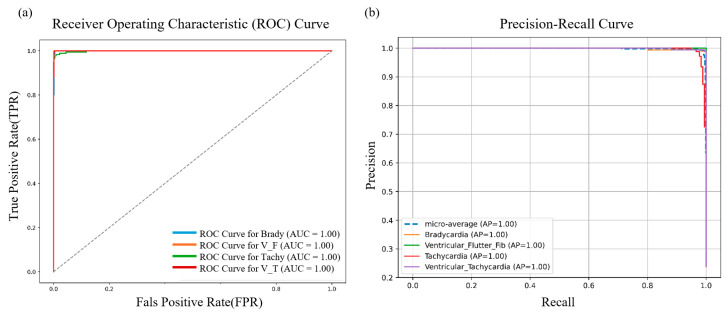
Performance evaluation of the Fusion-DMA-Net and ablation models: (**a**) Receiver Operating Characteristic (ROC) Curve. The diagonal dashed line represents the performance of a random classifier (AUC = 0.5). (**b**) Multiclass Precision–Recall (PR) curves with micro-averaged AP.

As shown in Figure 5, the class-wise ROC AUC scores for Bradycardia, Ventricular Flutter/Fibrillation, Tachycardia, and Ventricular Tachycardia are all close to 1.00, with a micro-average AUC of 0.995. The ROC curves closely follow the top-left corner, indicating that the model maintains an almost 100% true positive rate with an extremely low false positive rate across all threshold settings—reflecting high sensitivity and specificity. The Precision–Recall (PR) curves demonstrate similarly strong performance, with a micro-average precision (mAP) of approximately 0.998. Both macro- and micro-averaged precision and recall exceed 0.99, highlighting the model’s exceptional discriminative capability across all four arrhythmia categories. This indicates an extremely low likelihood of misclassification, particularly for high-risk rhythms such as ventricular flutter/fibrillation (V_F) and ventricular tachycardia (V_T), where the model shows outstanding reliability.

#### 4.2.3. Detailed Evaluation Metrics

To provide a more comprehensive view of the model’s classification capabilities for each arrhythmia class, Table 2 reports the detailed metrics achieved by Fusion-DMA-Net on the test set, including precision, sensitivity, specificity, F1-score, individual class accuracy, and overall accuracy.

As shown in Table 2, the Fusion-DMA-Net model demonstrates excellent classification performance across all four arrhythmia types. In particular, for Bradycardia, Ventricular Flutter/Fibrillation (VF), and Ventricular Tachycardia (VT), both precision and recall reach or approach 100%, with corresponding F1-scores exceeding 99.0%. These results reflect the model’s high sensitivity and specificity in detecting these rhythm types. Notably, the VT category achieves a perfect score in all metrics (100%), indicating that its spectral and rhythm features are highly distinctive and consistently identifiable by the model. In contrast, the Tachycardia class maintains a perfect precision of 100% but shows a slightly reduced recall of 96.00%, resulting in an F1-score of 97.96%. This may be attributed to subtle short-term morphological or rhythmic similarities between Tachycardia and VF waveforms, which could lead to occasional misclassifications.

From an overall perspective, the model achieves average precision, recall, specificity, and F1-score all exceeding 99.0%, with an overall accuracy of 99.05%. These results confirm that Fusion-DMA-Net can effectively extract discriminative temporal and spectral features directly from 10 s raw PPG segments in an end-to-end manner, without relying on handcrafted features. Even under class imbalance or when inter-class boundaries are ambiguous, the model maintains high recall (>96%) and precision (>99%), validating the effectiveness of its cross-scale residual attention and bidirectional sequential modeling components for robust, multi-class arrhythmia recognition.

### 4.3. Ablation Study

To assess the contribution of each module in the proposed Fusion-DMA-Net architecture, we designed seven systematically controlled ablation experiments, ensuring comparable model size in terms of parameter count and FLOPs. Under consistent conditions—using the same dataset, hardware environment, training hyperparameters, and keeping all other variables fixed—we conducted targeted module-wise ablation by selectively removing or adding specific components. These included the time-domain residual structure, the interactive attention module, the frequency-domain branch integration strategy, and the fusion mechanism. In each experiment, only one architectural element was modified at a time, while the rest of the model remained unchanged, enabling a step-by-step performance evaluation with strong interpretability. All experiments were conducted using 5-fold cross-validation with variance reporting to minimize the impact of data splitting bias. Model latency and resource consumption were also evaluated across different branches. In addition, ablation studies were performed to observe how the model’s performance changes when specific modules are incrementally removed or added. Detailed results and configurations can be found in the Supplementary Materials. The summary of results is presented in Table 3.

- Model 1: A CNN + BiLSTM baseline using time-domain input. This configuration consists of multiple 1D convolution layers (Conv1D), Batch Normalization, and MaxPooling layers for extracting local waveform features, followed by a bidirectional LSTM module for capturing global temporal dependencies. The output is flattened and passed through fully connected layers for classification.
- Model 2: Add Residual Branch. Based on Model 1, skip-connections were introduced within each convolutional module to form residual branches. This design aims to enhance feature propagation in deeper layers and improve the overall stability of the network. This model retains the original CNN + BiLSTM backbone without incorporating any attention mechanisms or frequency-domain information. By comparing its performance with that of the baseline model, the independent contribution of the residual structure within the time-domain branch can be effectively assessed.
- Model 3: Add CSRA Module. Building upon Model 2, this configuration further introduces the Cross-Scale Residual Attention (CSRA) mechanism, which comprises: ① Multi-Head Attention along the temporal dimension to capture long-range dependencies, and ② Channel Attention to emphasize critical sub-waveform features across channels. The CSRA module facilitates cross-scale feature fusion and adaptive weighting, thereby enhancing the discriminative power of time-domain representations while suppressing irrelevant or redundant features.
- Model 4: Frequency Branch Only. For the frequency-domain pathway, each 10 s PPG segment is processed by first computing the power spectral density (PSD) using the Welch method, followed by logarithmic normalization to enhance the dynamic range. A self-similarity matrix (SSM) is then constructed and fed into a Transformer encoder to extract global spectral structural features. These features are subsequently downsampled via a BiLSTM layer to achieve temporal alignment with the time-domain representations. Finally, the combined features pass through a fully connected layer with Dropout regularization and are fed into a softmax classifier to produce the final prediction output.
- Model 5: Add Frequency Branch (No Cross-Attention). Building upon the complete time-domain branch (including both residual and attention modules), this model incorporates an additional frequency-domain branch, identical in structure to Model 4. Both branches independently extract features and apply BiLSTM-based downsampling. The resulting representations are then simply concatenated before classification, without the use of any cross-attention mechanisms. This configuration is designed to evaluate the effectiveness of directly introducing frequency-domain information without inter-branch interaction.
- Model 6: Add the Hybrid Attention Module. Building on Model 5, we further introduce a comprehensive time–frequency fusion attention mechanism comprising: ① Channel Attention (CA) to reweight the concatenated multi-scale features by importance; ② Time–Frequency Cross-Attention to explicitly model inter-modal interactions between the two branches; and ③ Self-Attention combined with a Gated Fusion module to regulate the strength of the final fused representation. This design deepens the fusion process and strengthens cross-modal interplay, thereby enhancing discriminability prior to classification.
- Model 7: Fusion-DMA-Net (final model). We integrate all proposed components into a single architecture: a multi-layer residual time-domain backbone; frequency-domain spectrogram modeling; self-similarity–based structural encoding with a Transformer; and a tri-attention fusion scheme (channel, cross-modal, and self-attention) coupled with gated fusion. This full Fusion-DMA-Net serves as our final reference model and delivers the best overall performance in terms of accuracy, stability, and deployment efficiency.

As shown in Table 3, the performance of the proposed model alongside six comparative models, reporting average precision (Pre), sensitivity (Sen), specificity (Spe), F1-score, and overall accuracy (Acc) on the test set. When relying solely on a single-path time-domain architecture, the model achieves an accuracy of 87.09% and an AUC of 98.21%, representing the lower performance bound in the ablation study. With the introduction of residual connections (Model 2), the model exhibits notable improvements across all evaluation metrics, with accuracy rising to 90.72%, indicating that the residual structure facilitates deep feature propagation and contributes to stable convergence. Upon further integrating the Cross-Scale Residual Attention (CSRA) module (Model 3), the F1-score increases from 90.63% to 90.94%, and the AUC improves to 98.38%. These results suggest that the time-domain attention mechanism effectively enhances the model’s sensitivity to critical rhythm regions. When using the frequency-domain branch alone (Model 4), performance drops significantly, with an F1-score of only 76.76% and an accuracy of 76.73%. However, these results still exceed those of a random classifier, indicating that spectral structures contain meaningful discriminative information. After incorporating the frequency-domain branch into the overall architecture (Model 5), performance improves markedly to an F1-score and accuracy of 97.33%. Further enhancement is observed with the introduction of cross-modal attention mechanisms (Model 6), where the F1-score rises to 97.98%. This demonstrates that simple feature concatenation is insufficient to fully exploit frequency-domain information, and that cross-domain modeling provides substantial performance gains. The final architecture, Fusion-DMA-Net, achieves the best performance across all evaluation metrics, with an accuracy of 98.71%, F1-score of 98.74%, and AUC of 99.86%. These results validate the effectiveness of the multi-branch collaborative modeling and multi-level attention fusion strategy.

Table 4 further presents the parameter size, computational cost (MACs/FLOPs), and average inference latency per input sample for each model. Under a comparable overall parameter budget (approximately 1.46 M for all models), the introduction of additional modules leads to a moderate increase in latency. Specifically, the full Fusion-DMA-Net model exhibits an average inference latency of 30.5 ms, representing a slight increase of 24.8 ms compared to the baseline. However, this latency remains well within the real-time processing threshold (<100 ms), achieving an effective trade-off between computational efficiency and performance gains.

### 4.4. Comparative Study

To comprehensively evaluate the practical performance of the proposed model, this study conducts a comprehensive comparison between Fusion-DMA-Net and several existing approaches from two distinct perspectives. First, to further investigate the feasibility of accurately classifying arrhythmias using a single non-invasive signal modality—PPG—we present in Table 5 a comparative summary of related studies that utilized the same dataset (PhysioNet Challenge 2015) and employed either partially or fully overlapping signal sources. A horizontal comparison is conducted across models based on their sensitivity in detecting four distinct arrhythmic rhythms: Bradycardia (Brady), Tachycardia (Tachy), Ventricular Fibrillation (VF), and Ventricular Tachycardia (VT). It is important to note that some of the studies included in Table 5 were conducted under the task setting of “Reducing False Arrhythmia Alarms”, where the primary objective was to identify false alarms in ICU monitoring systems. As a result, these models were trained on mixed datasets containing both true positive and false positive alarm events. In contrast, the present study focuses specifically on the supervised classification of four types of arrhythmia using only true positive PPG alarm data, representing a more clearly defined and clinically relevant classification task. Nevertheless, we adopt sensitivity as the primary metric for horizontal comparison, as it is independent of the proportion of negative samples and thus offers a consistent basis for evaluating each model’s ability to detect specific arrhythmic events. Even though the underlying task structures may differ, sensitivity remains a reliable proxy for comparing recognition capability across methods, reflecting each approach’s effectiveness in identifying critical pathological rhythms within the same arrhythmia categories.

In the sensitivity evaluation across different arrhythmic rhythms, it is evident that under the constraint of using only PPG signals, Fusion-DMA-Net achieves exceptionally high sensitivity across all four arrhythmia types. Notably, it attains a 100% recall rate for both Ventricular Fibrillation (VF) and Ventricular Tachycardia (VT), markedly outperforming the majority of existing studies. In contrast, several prior works report VT recall rates ranging only from 49% to 90%, highlighting the limitations of traditional approaches in achieving accurate rhythm classification, particularly for high-risk arrhythmias. Therefore, leveraging the non-invasive nature of PPG signals alone holds significant potential to enhance the detection and monitoring of cardiac arrhythmias. Subsequently, given the relative scarcity of classification studies focused specifically on the four life-threatening arrhythmias—Bradycardia, Tachycardia, Ventricular Tachycardia (VT), and Ventricular Flutter/Fibrillation (VFB)—using PPG signals, we designed multiple comparative experiments to thoroughly validate the effectiveness and advantages of the proposed Fusion-DMA-Net model. All experiments were conducted under consistent data preprocessing protocols to ensure fair and reliable evaluation. Specifically, we adopted the exact same training/validation/test splits, input signal length (10 s), preprocessing procedures, class label definitions, and evaluation metrics as used in the main model to ensure fair comparison. In addition to benchmarking against existing studies, we also reproduced and evaluated three representative deep neural network architectures—AlexNet [39], VGG16 [40], and ResNet18 [41]—as baseline models. Furthermore, to explore the adaptability of traditional shallow classifiers for this task, we tested four classical machine learning models commonly used in previous studies: Decision Tree (DT), K-Nearest Neighbors (KNN), Support Vector Machine (SVM), and Ensemble Learning. The experimental results are summarized in Table 6.

As shown in Table 6, in terms of overall accuracy, the classical deep learning model VGG16 performed the worst, achieving only 71.63%. AlexNet and ResNet18 achieved accuracies of 80.54% and 89.57%, respectively—both clearly lower than those of traditional machine learning models such as KNN (98.4%), SVM (98.0%), and Ensemble methods (98.0%). In contrast, the proposed Fusion-DMA-Net achieved the highest overall accuracy of 99.05%, outperforming the majority of baseline and comparative methods across the board. In terms of detecting critical arrhythmias, Fusion-DMA-Net demonstrated outstanding performance, achieving over 98% precision and 100% recall for Bradycardia and Ventricular Fibrillation (VF). Notably, for Ventricular Tachycardia (VT), the model attained a perfect score—100% precision, recall, and specificity—highlighting its exceptional capability in identifying life-threatening rhythms with high reliability. In comparison, while ResNet18 shows acceptable performance in certain categories such as Tachycardia and Ventricular Fibrillation (VF), it still exhibits notable shortcomings in detecting Bradycardia (91.30% recall) and Ventricular Tachycardia (VT) (84.98% recall). VGG16 performs even worse, with VT recall dropping sharply to 53.99%, indicating imbalanced and unstable overall performance. AlexNet faces similar limitations, achieving only 72.73% recall on the VT class, highlighting the restricted generalization capability of its architecture when applied to PPG signal classification. In terms of machine learning methods, KNN and SVM outperform some deep learning models on average yet still exhibit shortcomings in specific arrhythmia categories. For instance, while SVM achieves a high recall of 99.8% for Tachycardia, its precision drops to 91.0%, indicating a higher false positive rate. Meanwhile, the Decision Tree model records a recall of only 82.0% for Ventricular Fibrillation (VF), reflecting a greater risk of misclassification when dealing with complex rhythm patterns. In terms of specificity, Fusion-DMA-Net also demonstrates exceptional stability, with all arrhythmia categories achieving values above 99.25%, and Ventricular Tachycardia (VT) reaching a perfect 100%. In contrast, other methods generally exhibit specificity levels in the 95–99% range, indicating a non-negligible risk of false positives. Notably, VGG16 shows particularly poor performance on Bradycardia, with a specificity of only 89.71%, further underscoring its limited robustness in distinguishing non-target signals.

These findings clearly demonstrate that the proposed model outperforms existing methods across all key evaluation metrics—including accuracy, sensitivity, and specificity. This strongly validates the effectiveness and robustness of the model’s synergistic architecture, which integrates multi-branch time–frequency fusion, cross-scale residual interactive attention, and bidirectional LSTM, in the context of four-class arrhythmia detection based on PPG signals.

### 4.5. Latency and Resource Consumption Analysis

To evaluate the deployment potential of the model on resource-constrained platforms such as wearable devices, we conducted a systematic inference performance benchmark of the trained model. The testing was performed using TensorFlow 2.15 on an NVIDIA RTX 5080 Laptop GPU (manufactured by NVIDIA Corporation, Santa Clara, CA, USA), with inputs consisting of both time-domain and frequency-domain features derived from PPG signals. The model contains a total of 1,465,175 parameters, occupying approximately 5.59 MB of memory using 32-bit floating-point precision.

As shown in Table 7, under a batch size of 1 (i.e., single-sample online inference mode), the model achieves a median latency of 27.46 ms and a 95th percentile (P95) latency of 36.41 ms, indicating that 95% of inference requests are completed within approximately 36 ms. This falls well within the commonly accepted “near real-time” latency range of 30–50 ms. As the batch size increases, the per-sample latency decreases significantly, reaching as low as 1.55 ms per sample at batch size 32, making the model highly suitable for high-throughput offline processing or cloud-based deployment scenarios. Compared to many mainstream devices, the model exhibits low memory consumption, with a maximum usage of only 16 MB, which is significantly below the typical memory limits supported by mobile and wearable platforms (e.g., Apple, manufactured by Apple Inc., Cupertino, CA, USA; Watch ≈ 128 MB RAM; embedded SoCs typically range from 512 MB to 2 GB). Interestingly, memory usage decreases with increasing batch size, indicating efficient memory management and further reinforcing the model’s deployment potential. However, it is important to note that the model’s frequency-domain branch relies on Welch PSD preprocessing, which—although it can be precomputed offline or implemented on low-power DSPs—still requires careful consideration of its computational overhead and power consumption during real-world deployment, particularly in edge or wearable scenarios.

## 5. Conclusions

In this study, we proposed Fusion-DMA-Net, a multi-scale attention-based deep learning model that fuses time- and frequency-domain features for the automatic detection of multiple types of arrhythmias using photoplethysmography (PPG) signals. The model integrates cross-scale residual attention mechanisms, self-similarity matrix modeling in the frequency domain, and multi-level attention fusion strategies, which collectively enhance its capability to model complex rhythm patterns and improve robustness against noise—all while maintaining an efficient network structure. The proposed framework explicitly captures the complementary information between temporal and spectral domains by designing separate feature extraction pathways and applying cross-attention and gating mechanisms for adaptive feature fusion. This enables the model to precisely extract and represent clinically relevant diagnostic features from raw PPG data. Experimental results demonstrate that Fusion-DMA-Net achieves an overall classification accuracy of 99.05%, with per-class precision, recall, and specificity all exceeding 98%, outperforming traditional machine learning methods and several existing deep learning approaches. Particularly for challenging rhythm types such as ventricular flutter (VF) and ventricular tachycardia (VT), the model exhibits superior discrimination and generalization capabilities. This study presents a novel architectural framework for arrhythmia detection based on PPG signals, offering a new design perspective for physiological signal analysis and providing a possibility for the future development of high-precision, low-cost intelligent wearable health monitoring systems.

Despite these promising results, several limitations remain. First, due to the limitations in dataset size and validation scope, the current study is based solely on four types of life-threatening arrhythmias, which restricts the model’s generalizability and limits its applicability in broader real-world scenarios, and the model’s generalizability across larger and more diverse populations requires further validation. Secondly, noise and artifact handling remains to be improved. The current study does not fully address motion artifacts and multi-source interferences commonly present in real-world PPG signals. The model has not yet been validated under conditions typical of wearable heart rate monitoring devices, such as motion states, low-quality signals, or dynamic environments. Third, real-time deployment has yet to be achieved—the model has not been implemented or evaluated on embedded platforms, and its practicality in edge computing scenarios remains unverified. Future research will focus on the following directions: expanding the dataset in both size and diversity, extending the model to cover lower-risk arrhythmia types, and evaluating its generalization capability across different datasets. We aim to explore the full spectrum of challenges, including false alarms and asystole detection. In addition, we will integrate signal enhancement and denoising techniques to improve the model’s robustness to signal degradation in dynamic or real-world scenarios. Further efforts will also be made to optimize the model architecture and parameter efficiency, with the goal of enabling deployment of wearable devices and developing lightweight arrhythmia detection models suitable for embedded and resource-constrained platforms.

## Figures and Tables

**Table 1:** Definitions of the five arrhythmia alarm types in the PhysioNet Challenge 2015.

Type	Explanation
Asystole	No QRS for at least 4 s
Extreme Bradycardia	Heart rate lower than 40 bpm for 5 consecutive beats
Extreme Tachycardia	Heart rate higher than 140 bpm for 17 consecutive beats
Ventricular Tachycardia	5 or more ventricular beats with heart rate higher than 100 bpm
Ventricular Flutter/Fibrillation	Fibrillatory, flutter, or oscillatory waveform for at least 4 s

**Table 2:** Detailed evaluation metrics of Fusion-DMA-Net on the test set.

Arrhythmia	Pre (%)	Sen (%)	Spe (%)	F1-Score	Acc (%)
Brady	98.41	100.00	99.45	99.20	99.59
Tachy	100.00	96.00	100.00	97.96	99.05
VF	98.02	100.00	99.25	99.00	99.46
VT	100.00	100.00	100.00	100.00	100.00
Overall	99.06	99.04	99.07	99.04	99.05

**Table 3:** Summary of Ablation Experiment Results.

Models	Pre (%)	Sen (%)	Spe (%)	F1-Score	AUC	Acc (%)
CNN + BILSTM	87.06 ± 0.91	87.61 ± 0.93	97.01 ± 0.31	86.88 ± 0.88	98.21 ± 0.27	87.09 ± 0.90
Add Residual Branch	90.84 ± 0.78	91.07 ± 0.78	96.9 ± 0.27	90.63 ± 0.78	97.99 ± 0.14	90.7 ± 0.77
Add CSRA Module	91.16 ± 0.59	91.40 ± 0.56	97.01 ± 0.20	90.94 ± 0.64	98.38 ± 0.26	91.01 ± 0.62
Frequency Branch Only	77.70 ± 0.80	77.41 ± 1.04	87.79 ± 0.36	76.76 ± 0.99	85.85 ± 0.54	76.73 ± 1.07
Add Frequency Branch (No Cross-Attention)	97.35 ± 0.43	97.27 ± 0.44	99.11 ± 0.14	97.33 ± 0.43	99.84 ± 0.13	97.33 ± 0.43
Add the hybrid attention module	97.99 ± 0.68	97.94 ± 0.70	99.33 ± 0.23	97.98 ± 0.68	99.87 ± 0.14	97.99 ± 0.68
Fusion-DMA-Net (Baseline)	98.85 ± 0.68	98.72 ± 0.43	98.94 ± 0.35	98.7 ± 0.47	99.86 ± 0.12	98.71 ± 0.45

**Table 4:** Comparison of Parameter Size, Computational Complexity, and Inference Latency across Different Model Variants.

Models	Params (Mean)	MACs	FLOPs	Latency (ms)
CNN + BILSTM	1,452,499	93,411,096	186,822,192	5.72
Add Residual Branch	1,474,882	219,808,028	439,616,056	27.61
Add CSRA Module	1,461,904	184,783,547	369,567,094	31.88
Frequency Branch Only	1,465,426	147,491,088	294,982,176	22.46
Add Frequency Branch (No Cross-Attention)	1,469,326	189,256,451	378,512,902	30.04
Add the hybrid attention module	1,461,904	184,783,547	369,567,094	30.91
Fusion-DMA-Net (Baseline)	1,461,904	184,783,547	369,567,094	30.5

**Table 5:** Comparison of sensitivity across studies based on PhysioNet Challenge 2015 dataset.

Reference	Signal Type	Sensitivity			
		Brady	Tachy	VF	VT
Antink and Leonhardt [34]	ECG, PPG, ABP	100%	100%	67%	90%
Eerikanen et al. [35]	ECG, PPG, ABP	96%	99%	75%	84%
Kalidas and Tamil [36]	ECG, PPG	100%	100%	100%	84%
Caballero and Mirsky [37]	PPG, ABP	95%	97%	89%	49%
Paradkar and Chowdhury [38]	PPG	88.80%	97.60%	50%	88.10%
Qananwah et al. [16]	PPG	98.30%	93%	92.10%	99.70%
Current study	PPG	100%	96%	100%	100%

**Table 6:** Comparative performance metrics of previous methods.

Models	Arrhythmia	Pre (%)	Sen (%)	Spe (%)	Acc (%)
DT	Brady	99.8	98.5	93.3	93.1
	Tachy	96.8	85.0	85.8	
	VF	98.1	82.0	85.2	
	VT	93.3	96.6	95.7	
KNN	Brady	100	98.3	100	98.4
	Tachy	98.9	95	99.7	
	VF	98.3	96.8	99.8	
	VT	97.9	99.7	96.8	
SVM	Brady	98.3	98.3	99.8	98.0
	Tachy	98.9	91	99.8	
	VF	98.3	96.8	99.8	
	VT	96.5	100	94.5	
Ensemble	Brady	98.3	100	99.8	98.0
	Tachy	100	93	100	
	VF	98.3	93.7	99.8	
	VT	97.3	100	95.9	
AlexNet	Brady	79.79	80.65	93.08	80.54
	Tachy	75.56	77.71	92.14	
	VF	86.41	89.90	94.79	
	VT	79.50	72.73	94.10	
VGG 16	Brady	71.26	79.60	89.71	71.63
	Tachy	65.85	63.13	87.57	
	VF	87.46	93.06	96.06	
	VT	61.68	53.99	88.57	
ResNet 18	Brady	95.62	91.30	98.66	89.57
	Tachy	87.18	85.25	95.24	
	VF	89.01	98.04	96.42	
	VT	87.07	84.98	95.70	
Fusion-DMA-Net	Brady	98.41	100	99.45	99.05
	Tachy	100.0	96.0	100.0	
	VF	98.02	100.0	99.25	
	VT	100.0	100.0	100.0	

**Table 7:** Inference Latency, Throughput, and GPU Memory Usage under Different Batch Sizes.

Batch Size	P50 (ms)	P95 (ms)	P99 (ms)	Per-Sample (ms)	Throughput (Samples/s)	GPU Mem Used (MB)
**1**	27.5	36.4	39.2	27.5	35.7	15.9
**8**	26.3	35.2	37.4	3.3	294.1	15.9
**16**	28.6	38.8	39.5	1.8	551.7	6.3
**32**	48.9	67.2	71.3	1.55	643.6	6.4

## Data Availability

The original data presented in the study are openly available in PhysioNet at https://physionet.org/content/challenge-2015/1.0.0/ (accessed on 24 September 2025).

## Supplementary Materials

The following supporting information can be downloaded at https://www.mdpi.com/article/10.3390/s25195985/s1; Figure S1: Performance evaluation of the Fusion-DMA-Net and ablation models: (a) CNN + BiLSTM model; (b) Model with attention mechanism and residual blocks; (c) Confusion matrix for the frequency-domain branch; (d) Model with added frequency-domain branch (final version); Table S1: Summary of ablation experiment results.
