# Supplementary material for: Multi-Classification Model for PPG Signal Arrhythmia Based on Time–Frequency Dual-Domain Attention Fusion
Source: Sensors (Basel). 2025 Sep 27;25(19):5985. doi: 10.3390/s25195985 (PMC12526570; doi:10.3390/s25195985)
Supplement: Supplementary file 1 [file sensors-25-05985-s001.zip › sensors-3850166-supplementary.pdf]

Supplementary materials

1. Ablation Study

To evaluate the contribution of each component in the proposed Fusion-DMA-Net, a set of systematic ablation experiments was designed to observe how the model's performance changes when specific modules are incrementally removed or added. Under a consistent dataset, hardware environment, and hyperparameter configura-tion, specific components were incrementally added or removed to evaluate their indi-vidual impact on model performance. All experiments adopted five-fold cross-validation to mitigate sampling bias. The summary of results is presented in Table 3, and the corresponding confusion matrices are shown in Figure S1(a-d).

- Model 1: A CNN + BiLSTM baseline using time-domain input. This configuration consists of multiple 1D convolution layers (Conv1D), Batch Normalization, and MaxPooling layers for extracting local waveform features, followed by a bidirectional LSTM module for capturing global temporal dependencies. The output is flattened and passed through fully connected layers for classification.
- Model 2: Built upon Model 1 by introducing residual connections and interactive residual attention mechanisms. Specifically, skip-connections are added to each convolutional block, and multi-head attention is applied along both temporal and channel dimensions to enhance discriminative sub-waveform responses while suppressing irrelevant or redundant features.
- Model 3: integrates frequency-domain features by combining the arctangent of frequency signals with a self-similarity matrix (SSM) processed via a Transformer. Specifically, each 10-second PPG segment undergoes power spectral density (PSD) estimation using the Welch method, followed by logarithmic normalization to enhance dynamic range. An SSM is then constructed from the normalized PSD and fed into a Transformer encoder to extract global spectral structure features. These are subsequently downsampled using a BiLSTM to align with time-domain features. Finally, the combined features pass through a fully connected layer with dropout regularization and are classified using a softmax output layer..
- Model 4: extends the time-domain branch by introducing a parallel frequency-domain branch. After processing, the frequency-domain features are also downsampled via BiLSTM and aligned with time-domain representations. The concatenated features are then used for final classification.
- Model 5: Represents the complete proposed Fusion-DMA-Net, which integrates a multi-level fusion strategy on top of the combined time-frequency representations. This includes channel attention, cross-attention, self-attention, and a gating mechanism that performs importance reweighting, domain interaction, and adaptive integration strength control across modalities. This configuration constitutes the final architecture.

Table S1. Summary of Ablation Experiment Results

Models	Pre (%)	Sen (%)	Spe (%)	F1-score	Acc (%)
**Fusion-DMA-Net**	**98.41**	**100.00**	**99.45**	**99.20**	**99.05**
CNN + BILSTM	91.52	93.19	91.52	92.22	92.95
Adding attention mechanism and residual blocks	93.72	93.64	97.82	93.65	93.64
frequency domain branch	82.48	82.04	82.5	82.07	82.04
Add the frequency domain branch	97.99	97.96	99.3	97.95	97.96

As shown in Table S1, the proposed model and its three ablated variants were evaluated in terms of average precision (Pre), sensitivity (Sen), specificity (Spe), F1-score, and overall accuracy (Acc) on the test set. For Model 1 (CNN + BiLSTM), the overall accuracy reached 92.95%, but considerable false positives were observed in certain rhythm types. For example, premature ventricular contractions (PVC) were misclassified as premature atrial contractions (PAC) at a rate of 4.08%, and ventricular tachycardia (VT) was confused with supraventricular tachycardia (SVT) at a rate of 3.61%. These results indicate that relying solely on time-domain convolution and LSTM-based modeling is insufficient for distinguishing rhythm types with highly similar waveform morphologies. With the introduction of interactive residual attention mechanisms in Model 2, the performance improved significantly. Specifically, specificity increased from 91.52% to 97.82%, precision rose from 91.52% to 93.72%, and the F1-score improved from 92.22% to 93.65%. This confirms that the attention modules play a crucial role in suppressing false positives by compensating for CNN's limited sensitivity to fine-grained waveform variations in the time domain. Building upon Model 2, Model 3 added a frequency-domain branch. The model achieved precision and recall of 97.99% and 97.96%, respectively, with an F1-score of 97.95%, and an overall accuracy of 97.96%. Notably, for the VF (ventricular fibrillation) category, the recall reached 100%, and mutual misclassification between VT and SVT dropped to 0.41%. These findings highlight that frequency-domain representations effectively complement the time domain, particularly in capturing subtle oscillations and harmonic patterns.

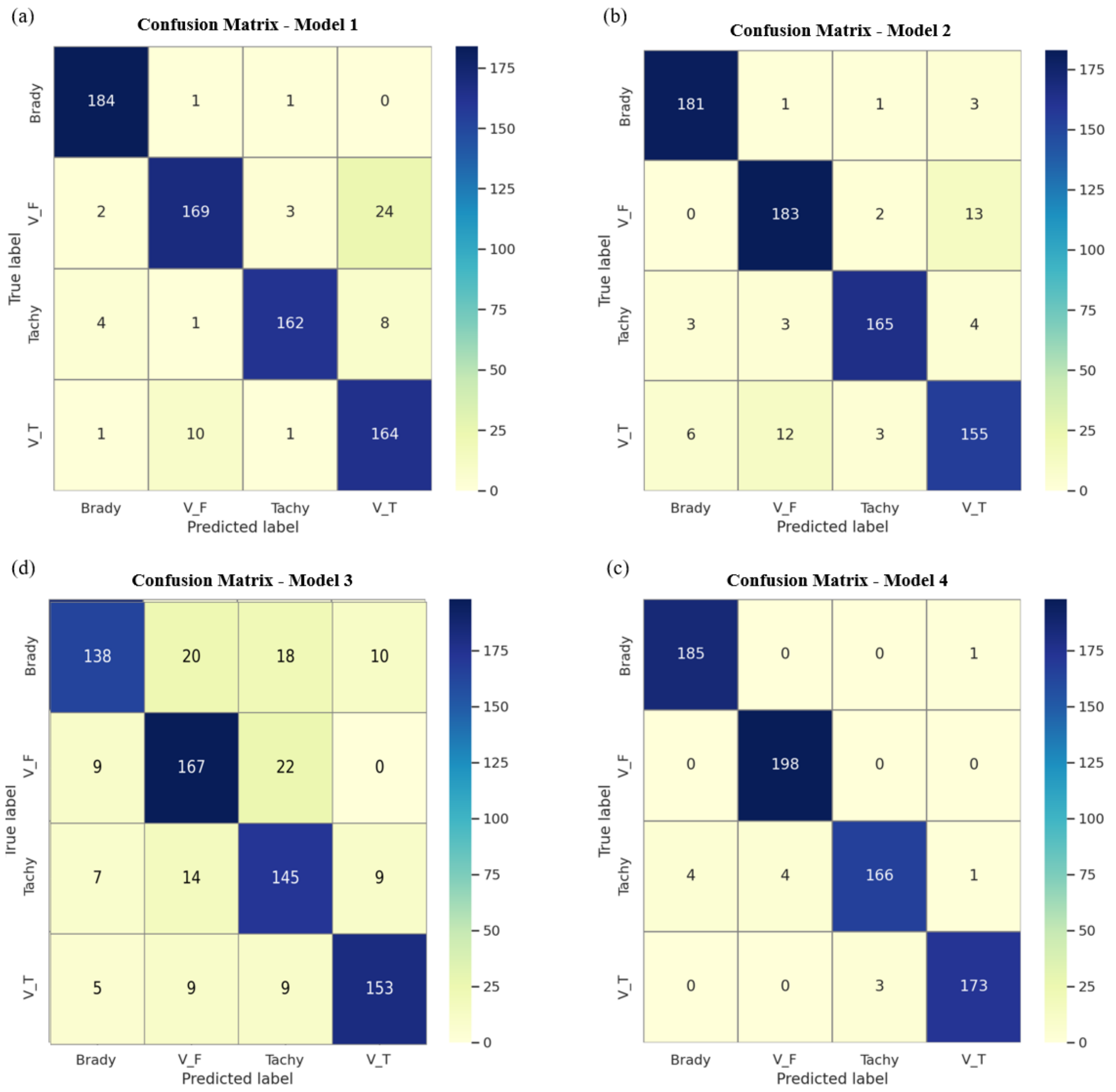

Figure S1. Performance evaluation of the Fusion-DMA-Net and ablation models: (a) CNN + BiLSTM model; (b) Model with attention mechanism and residual blocks; (c) Confusion matrix for the frequency-domain branch; (d) Model with added frequency-domain branch (final version).

Finally, the Fusion-DMA-Net architecture integrated all aforementioned improvements, adding channel attention, cross-attention, self-attention, and a gated fusion mechanism on top of the time-frequency concatenation. This allowed the model to perform adaptive, dynamic weighting and information integration across feature channels, temporal sequences, and modalities. As a result, the model achieved 98.41% precision, 100.00% recall, 99.45% specificity, 99.20% F1-score, and an overall accuracy of 99.05%—the best performance among all variants. These results strongly validate the effectiveness of multi-level attention mechanisms and gated fusion strategies in enhancing classification accuracy for complex rhythm disorders.

2. Welch Power Spectral Density (PSD) Parameters

The frequency-domain branch relies on Welch's method to compute the power spectral density (PSD) of the PPG signals. The parameters used are:

- **Segment Length (nperseg)**: 512 samples
- **Overlap (noverlap)**: 256 samples
- **Window Type**: Hamming window (default in scipy.signal.welch)
- **Sampling Rate (fs)**: 250 Hz
- **Frequency Resolution**: ~0.488 Hz (calculated as fs / nperseg)
- **Output Size**: 257 frequency bins per segment (i.e., nperseg // 2 + 1)

These settings provide a balance between frequency resolution and temporal averaging, and are well-suited for characterizing arrhythmia-related frequency features in 10-second PPG segments.

---

3. SSM (Self-Similarity Matrix) and Normalization

The self-similarity matrix (SSM) is constructed using:

- $SSM = X \cdot X^{\mathrm{T}},$ where X is the PSD-transformed feature map per segment
- Before similarity computation, all PSD vectors are **log-normalized** via log1p() (i.e., log(1 + x)) to suppress skewed magnitudes
- After similarity computation, the SSM is **rescaled using min-max normalization** to the range [0, 1] for numerical stability and improved convergence in the transformer block.

This normalization ensures that dynamic range differences between classes or segments do not distort the self-attention mechanisms.
